# Hypermetabolic Ipsilateral Supraclavicular and Axillary Lymphadenopathy: Optimal Time Point for Performing an ^18^F-FDG PET/CT after COVID-19 Vaccination

**DOI:** 10.3390/diagnostics12123073

**Published:** 2022-12-06

**Authors:** Kwadwo Antwi, Federico Caobelli, Ken Kudura, Hans-Georg Buchholz, Martin Hoffmann, Mathias Schreckenberger

**Affiliations:** 1 Department of Nuclear Medicine, Clinic of Radiology and Nuclear Medicine, St. Claraspital Basel, 4058 Basel, Switzerland; 2 Department of Nuclear Medicine, Inselspital Bern, University Hospital Bern, 3010 Bern, Switzerland; 3 Department of Nuclear Medicine, University Hospital Mainz, 12459 Berlin, Germany

**Keywords:** hypermetabolic ipsilateral supraclavicular and axillary lymphadenopathy, HLA, COVID-19 vaccination, ^18^F-FDG PET/CT

## Abstract

Background: We aimed to evaluate the incidence of severe acute respiratory syndrome coronavirus type-2 (SARS-CoV2) vaccine-related hypermetabolic lymphadenopathy (HLA) and evaluate which time point produces the least number of false-positive findings in an ^18^F-2-Fluor-2-desoxy-D-glucose positron emission tomography/computed tomography (^18^F-FDG PET/CT). Methods: For this retrospective, multi-center imaging study, patients with any form of SARS-CoV2 vaccination prior to an ^18^F-FDG-PET/CT were included between January 2021 and December 2021. Patients were divided into six groups according to the time point of vaccination prior to their ^18^F-FDG-PET/CT imaging, e.g., group one (0–6 days) and group six (35–80 days). As the reference standards, the SUVmax of the mediastinal blood pool (MBP) and the SUVmax contralateral reference lymph node (RL) were determined. (A) The absolute SUVmax of HLA, (B) the ratio of SUVmaxHLA/SUVmax mediastinal blood pool (rHLA/MBP), (C) the ratio SUVmax HLA vs. SUVmax contralateral reference lymph node (rHLA/RL), (D) and the incidence of HLA defined as rHLA/MBP > 1.5 were assessed. Results: Group one (days 0–6) showed the highest incidence of HLA 16/23 (70%) and rHLA/MBP (2.58 ± 2.1). All three parameters for HLA reduced statistically significantly in the comparison of Groups 1–3 (days 0–20) versus Groups 4–6 (days 21–80) (*p*-values < 0.001). Conclusions: If feasible, an FDG PET should be postponed by at least 3 weeks after SARS-CoV2 vaccination, especially if an accurate evaluation of axillary status is required.

## 1. Introduction

Due to the ongoing global coronavirus disease 2019 (COVID-19) pandemic, the vaccination campaign against its aetiologic agent, the severe acute respiratory syndrome coronavirus type 2 (SARS-CoV2), has emerged worldwide with over 12 billion dosages applied (World Health Organization Interim statement on COVID-19 vaccination, 07/2022) [1]. Due to the ongoing mutation of the virus, further vaccinations will most likely be implemented by autumn 2022.

After the injection of the SARS-CoV2 vaccine into the deltoid muscle, a physiologic immunogenic clinical response occurs, leading to a reactive ipsilateral supraclavicular and axillary lymphadenopathy (HLA) [2,3,4,5,6,7]. This is a common finding after vaccination, being present in 0.3% of subjects receiving the BNT162b2 vaccine according to data from physical examination [8]. However, the sensitivity of a physical examination in detecting reactive lymphadenopathy is relatively low [8]; hence, the rate of ipsilateral axillary and supraclavicular lymphadenopathy after SARS-CoV2 vaccination is expected to be higher in a more sensitive imaging modality such as an ^18^F-Fluorodeoxyglucose (FDG) positron emission tomography/computed tomography (PET/CT) [6,9,10].

HLA may potentially lead to incorrect interpretation of ^18^F-FDG PET/CTs, especially in oncology patients, or may cause unnecessary procedures (e.g., biopsy) as hypermetabolic lymph nodes may be falsely considered malignant.

Prior ^18^F-FDG PET/CT studies performed after the H1N1 vaccination have shown that these findings usually resolve after 12–14 days but could persist up to 4–6 weeks after vaccination [11,12,13,14,15,16]. To date, only a few studies, case reports, or case series are available on the duration of HLA [17,18,19,20]. Therefore, there is a need for precise information about the incidence and intensity of SARS-CoV2 vaccine-related HLA, as well as the optimal time point at which an ^18^F-FDG PET/CT can be performed after SARS-CoV2 vaccination.

We, therefore, aimed to:

1. Evaluate the incidence of SARS-CoV2 vaccine-related axillary and supraclavicular HLA.
2. Evaluate which time point produces the least number of false-positive findings. HLA is expected to present with an intensive ^18^F-FDG uptake shortly after vaccination but decreases significantly after a certain time point.

## 2. Materials and Methods

### 2.1. Study Design and Patient Selection

For this retrospective, multi-center imaging study, oncological or non-oncological patients with any form of SARS-CoV2 vaccination (first, second, or third/booster shot) prior to an ^18^F-FDG-PET/CT between January 2021 and December 2021 were included. Data were collected from patients’ PET/CT images from the nuclear medicine department of St. Claraspital Basel, Switzerland and the nuclear medicine department of the University Hospital Mainz, Germany.

Clinical and imaging data included age, weight, gender, oncological or non-oncological diagnosis, date and sequence of vaccination, date of the ^18^F-FDG PET/CT imaging, injected activity of ^18^F-FDG, and the maximum standard uptake value (SUVmax) of HLA.

Inclusion criteria were: (1) patients who had undergone SARS-CoV2 vaccine injection in the deltoid region (left or right) prior to an ^18^F-FDG PET/CT scan and (2) age ≥ 18. Exclusion criteria were: (1) documented concomitant intramuscular vaccination in the deltoid region other than against SARS-CoV2, (2) vaccine injection in a non-deltoid region, (3) missing information on the site of vaccination, missing information of the exact date of vaccination or if the date of vaccination to the ^18^F-FDG PET/CT imaging was >80 days, and (4) known tumor involvement of axillary or supraclavicular lymph nodes in the side of injection. HLA was deemed as possibly malignant if there was (a) histological confirmation of malignancy, (b) metabolic or morphological progression on follow up imaging, and (c) HLA was located in a locoregional lymph node of the primary tumor or if the primary tumor showed extensive lymph node involvement in non-locoregional lymph nodes. These patients were excluded from the analysis.

This study was conducted in compliance with good clinical practice (GCP) rules and the Declaration of Helsinki. Patient records and information were de-identified before analysis. For patients from Basel, Switzerland, all patients gave informed consent to the use of their clinical and imaging data for research purposes, and the study was approved by the regional scientific ethics committee (EKNZ 2022-01174). For patients from Mainz, Germany, institutional review board approval was waived given that this study utilized the retrospective analysis of blinded clinical data.

Patients were divided into six groups according to the time point of vaccination to PET/CT imaging: group one (0–6 days), group two (7–13 days), group three (14–20 days), group four (21–27 days), group five (28–34 days), and group six (35–80 days). The median time between vaccination and the ^18^F-FDG PET/CT was 19 (IQR 7–34) days.

### 2.2. Patient Preparation and PET/CT Acquisition

All patients underwent an ^18^F-FDG PET/CT performed according to the institute’s clinical protocol. A fasting period of at least six hours before the radiopharmaceutical administration was required for all patients. ^18^F-FDG was injected at 2–3 MBq per kilogram body weight (82.4 ± 14.9 MBq, ranging from 43 to 106 MBq) after verification of blood glucose levels below 10 mmol/L (180 mg/dL).

PET/CT images were acquired on two different scanners in a supine position 60 min after FDG injection: a PET/16-detector CT scanner (Gemini^®^ TF 16 PET/CT Philips, Best, The Netherlands) and a PET/64-detector CT scanner (GE Discovery Molecular Insights—DMI PET/CT, GE Healthcare, Waukesha, WI, USA).

A low dose CT or contrast media enhanced CT (obtained from the vertex to either the inguinal region, mid-thighs, popliteal region, or feet) was acquired in all patients for attenuation correction and to provide anatomical correlation (matrix size 512 × 512, field of view 50 cm, and slice thickness 3.75 mm). This was immediately followed by the PET acquisition in five or six bed positions (patient’s size-adapted) with an acquisition time of 2.5 min/bed position (matrix size 256 × 256 and field of view 70 cm). Images were reconstructed with the ordered subset expectation maximization (OSEM, Gemini^®^ TF 16 PET/CT Philips, Best, The Netherlands) iterative algorithm, block sequential regularized expectation maximation (BSREM, GE Healthcare, Waukesha, WI), and TOF reconstruction with standardized parameters for oncological PET imaging. Calibration of PET scanners and their cross-calibration was performed using the IEC body phantom as follows. To enhance the comparability of data acquired on the two different PET/CT scanners, PET acquisition and reconstruction protocols, as proposed by the EARL-FDG-PET/CT accreditation program, were used. Conforming to EANM EARL guidelines, the six hollow spheres (diameters ranging from 10 to 37 mm) of the NEMA IEC body phantom were filled with FDG to achieve a radioactive concentration of approximately 20 kBq/mL. The large chamber (background) of the phantom was filled with 2 kBq/mL. Two bed positions were acquired for 5 min/bed. Reconstructed PET images were analyzed with a volume of interest (VOI) template to estimate SUV maximum based recovery coefficient (RC) for each sphere. Ratios of the RC (BSREM) to RC (Lor-ToF) were calculated and plotted as a function of the spheres’ diameters to define correction factors for upscaling SUV when acquired on the Philips scanner. SUVmax measurements using OSEM on the Gemini^®^ TF 16 PET/CT Philips were then corrected as follows: SUVmax corrected (=SUVmax(Philips) × correction factor(diameter)).

### 2.3. Image Interpretation

Images were retrospectively interpreted visually and semi-quantitatively by one physician with dual-board certification in nuclear medicine and radiology and having more than eight-years’ experience in PET/CT readings using the Sectra workstation (IDS7, Version 24.1.).

SUVmax was calculated as ^18^F-FDG uptake (kBq/mL) divided by the injected dose (MBq) and multiplied by the lean body weight (kg). SUVmax measurements were performed by manually placing a VOI on fused images around the most avid lymph nodes in the axillary levels or on the most avid supraclavicular lymph nodes ipsilateral side of the vaccination injection. SUVmax measurements were repeated on the most avid contralateral axillary level 1, 2, 3, or supraclavicular lymph nodes. In cases where the lymph nodes did not show any visible FDG uptake, SUVmax uptake was performed using the morphological CT appearance of the lymph node on fused images. As per the reference standards, the SUVmax of the axillary/supraclavicular lymph nodes contralateral to the side of vaccination (RL) and the SUVmax of the mediastinal blood pool (MBP), by placing a standard 1 cm^3^ VOI in the aortic arch, were recorded.

For all groups, the ratio SUVmaxHLA/SUVmaxMBP (rHLA/MBP) as well as the ratio SUVmax of HLA versus SUVmax reference lymph (rHLA/RL) node was calculated. For incidence calculation, lymph nodes with rHLA/MBP ≥ 1.5 were considered positive and rHLA/MBP < 1.5 were considered negative.

### 2.4. Statistical Analysis

Statistical analysis was performed using commercially available software (JMP 13.0, SAS, Cary, NC, USA). Categorical variables were reported as frequency and percentage. Continuous variables were evaluated for normal distribution and reported as means and standard deviation. Differences in SUVmaxHLA, rHLA/MBP, and rHLA/RL between all groups were compared by an independent t-test or a Mann–Whitney U Test if data was not normally distributed. Univariate one-way ANOVA was used to compare SUVmaxHLA and rHLA/MBP as well as rHLA/RL between the first, second, and third shot. For all comparisons, a *p*-value of <0.05 was considered statistically significant.

## 3. Results

### 3.1. Baseline Characteristics

Between January 2021 and December 2021, the clinical data from 2028 FDG PET/CTs were screened. Of all patients scanned between January 2021 and December 2021, 146 patients met all the inclusion criteria. An ^18^F-FDG PET/CT was performed as standard of care for oncological indications in most patients (144/146, 98.6%) (Table 1). The mean age was 66.39 ± 12.9 years with an equal distribution of females n = 72 (49%) and males n = 74 (51%). Most patients received their vaccination in the left deltoid muscle (n = 120, 82%). Of the included patients, 37/146 (25%) received the first shot, 73/146 (50%) received the second and 36/146 (25%) received their third one.

### 3.2. Evaluation of Absolute SUVmax HLA

**Figure 1:**
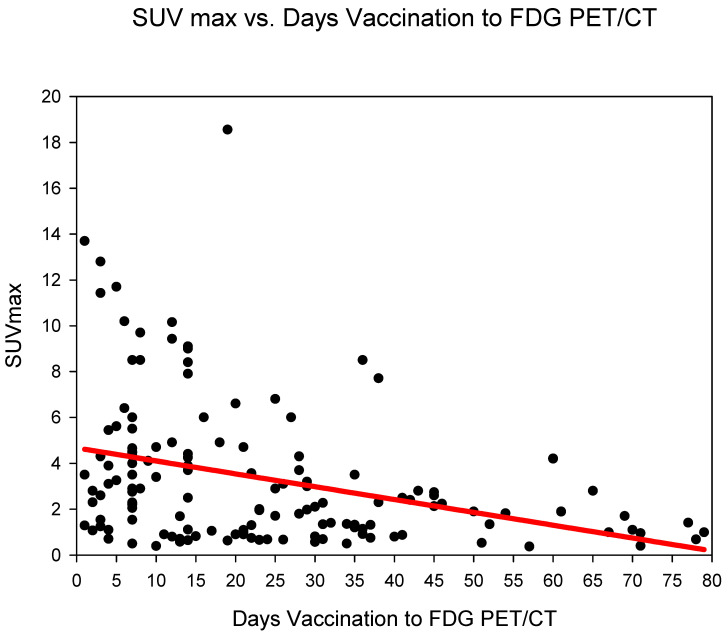
The absolute SUVmax (g/mL) of HLA (*y*-axis) against time (in days) from COVID-19 vaccination (*y*-axis). A steady decrease in SUVmax is observed.

Measurement of absolute SUVmax HLA showed the highest SUVmax in group three (days 14–20), SUVmax 5.05 ± 4.33 followed by group one (days 0–6) (SUVmax 4.97 ± 4.1) and group two (days 7–13) 3.9 ± 2.81. The SUVmax of HLA dropped markedly after 21 days (from group four) with values remaining at around 2.1–2.2 until group six (35–80 days) (Figure 1) (Table 2). SUVmax HLA, reduced statistically significantly (*p* < 0.001) in the comparison groups 1–3 (days 0–20) versus groups 4–6 (days 21–80) (Table 3).

### 3.3. Evaluation of rHLA/MBP

**Figure 2:**
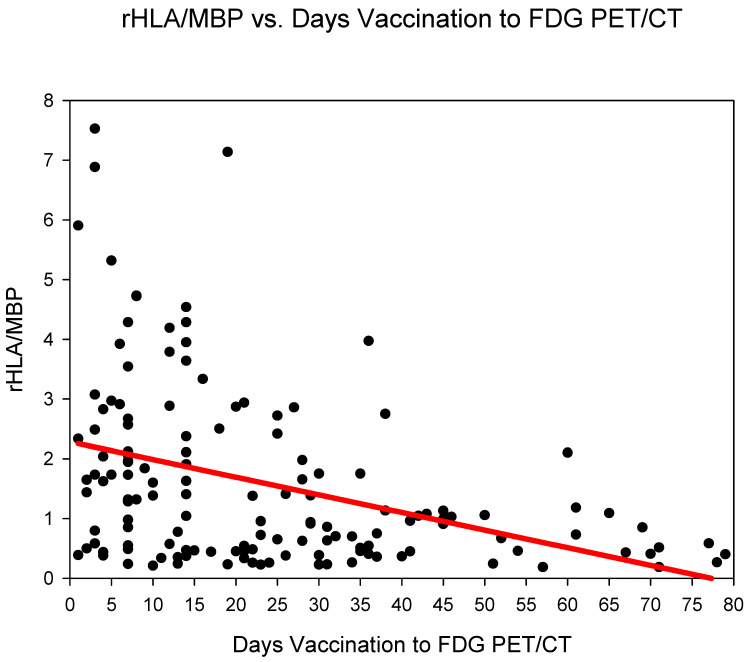
The ratio SUVmaxHLA/SUVmaxMBP (rHLA/MBP) plotted against time (days) from COVID-19 vaccination. A steady decrease in the rHLA/MBP with values approaching <1.5 after 21 days is observed.

Group one (days 0–6) showed the highest rHLA/MBP 2.58 ± 2.1, followed by group three (days 14–20) 2.23 ± 1.8 and group two (days 7–13) 1.83 ± 1.38 (Figure 2) (Table 2). The rHLA/MBP dropped markedly after 21 days post COVID-19 vaccination (from group four) with rHLA/MBP remaining at 1.07. rHLA/MBP reduced statistically significantly (*p* < 0.001) in the comparison groups 1–3 (days 0–20) versus groups 4–6 (days 21–80) (Table 3).

### 3.4. Evaluation of rHLA/RL

**Figure 3:**
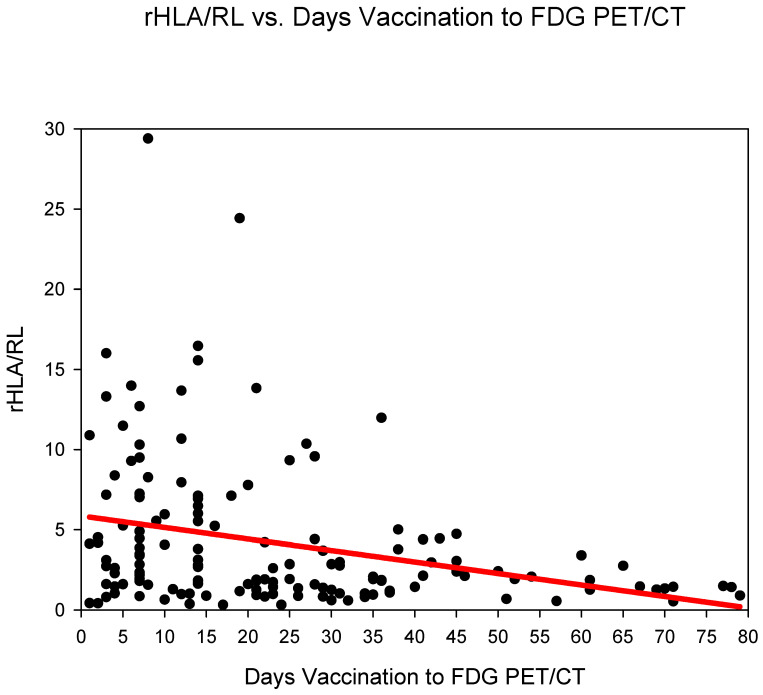
The ratio SUVmaxHLA/SUVmaxRL (rHLA/RL) plotted against time (days) from COVID-19 vaccination. A steady decrease in the rHLA/MBP with values approaching <1.5 after 21 days is observed.

Group three (days 14–20) showed the highest rHLA/RL 6.11 ± 5.99 followed by group one (days 0–6) 5.5 ± 4.82 and group two (days 7–13) 5.41 ± 5.73 (Figure 3) (Table 2). The rHLA/RL dropped markedly after 21 days post COVID-19 vaccination (from group four). rHLA/RL reduced statistically significantly (*p* < 0.001) in the comparison groups 1–3 (days 0–20) versus groups 4–6 (days 21–80) (Table 3).

### 3.5. Incidence of HLA According to Groups

Group one (days 0–6) showed the highest incidence 16/23 (70%) of HLA, followed by group three (days 14–20), 12/21 (57%) and group two (days 7–13), 14/32 (44%). The incidence of HLA dropped markedly from group four (days 21–27 post COVID-19 vaccination) with the lowest incidence found in group six (days 35–80), 4/36 (11%) (Table 4).

### 3.6. Evaluation of Vaccination Sequence

In all groups, HLA SUVmax was lowest in the third vaccination shot. (Table 5). There were no significant differences in SUVmax of HLA, rHLA/MBP, and rHLA/RL between the first, second, or third shot.

## 4. Discussion

SARS-CoV2 vaccination has emerged as an important factor to control the spread of the COVID-19 pandemic [21].

**Figure 4:**
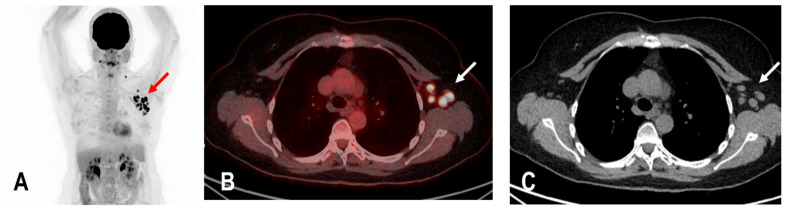
The maximum intensity projection (MIP) (**A**) of a patient with rectal carcinoma and HLA in the ipsilateral draining supraclavicular and axillary lymph nodes (red arrow) in the ^18^F-FDG PET/CT 10 days following COVID-19 vaccination. Fused images (**B**) show that the hypermetabolism is located in the moderately enlarged lymph nodes (white arrow) which still show a physiological fat hilus (SUVmax: 18.6, rHLA/MBP: 7.75, and rHLA/RL:11.6 (**C**)).

The widespread administration of such vaccines may pose diagnostic challenges in patients undergoing an ^18^F-FDG PET/CT as enlarged and hypermetabolic lymph nodes (HLA) following vaccination were frequently reported (Figure 4) [6,9,10].

In fact, a proportion of vaccine-derived spike proteins do not remain within the site of injection but rather migrate through lymphatic vessels reaching the proximal lymph nodes, wherein they also stimulate the production of the immunoglobulins G and A (IgG and IgA) [22]. While the amount of spike protein outside the site of injection is limited [23], it is still enough to elicit both a cellular (T-cell) and humoral (B-cell) immune response in the regional lymph nodes. Furthermore, mRNA vaccines sustain a more robust and rapid B-cell proliferation in the germinal center of the lymph node compared to protein-based vaccines or vaccines containing inactive or attenuated pathogens [24], which renders these vaccines to more likely cause regional lymphadenopathy.

This concept was supported by the demonstrated correlation between the amount of circulating SARS-CoV2 antibodies and the incidence of regional lymphadenopathy [4]. For example, a recent paper showed that there is a difference in the incidence and characteristics of HLA in patients receiving the first and those receiving the second vaccine shot, being more pronounced in the second case [4]. Furthermore, it should be noted that memory B- and T-cell responses are different after a first, second, and booster shot [25]. If memory cells have already undergone clonal expansion and affinity maturation as happens after the first shot, then the immune response after the second and third shot has no lag period.

This concept seems to be confirmed by our results. Although not reaching statistical significance, there is a tendency toward a reduction in the incidence of HLA after the third shot compared to that after the first and second shot.

Knowledge of the incidence and intensity of regional lymphadenopathies as well as the optimal time point after SARS-CoV2 vaccination to perform an FDG PET/CT is crucial.

A few papers could be found in the literature on this important topic. The above-mentioned paper by Cohen et al. [4] investigated the overall incidence of hypermetabolic lymph nodes after vaccination with the Pfizer vaccine and also its relevance to FDG PET/CT scan interpretation in oncologic patients. In their paper, the incidence of hypermetabolic lymph nodes was 36.4% in patients with a single vaccine shot and 53.9% after the second shot. The same authors further investigated the impact after a third vaccine shot and found that the incidence was slightly lower compared to that after the second shot (47.5%), probably due to the time interval between the second and third shot causing a reduced efficacy of the memory immune response compared to what was observed between the first and second shot [18]. However, the incidence increased up to 82.5% if images were acquired within 5 days of vaccination [18]. Our work expanded on this important topic.

To be able to transfer our study data for adequate clinical utilization, patients were grouped according to the time duration of the FDG PET/CT imaging to the date of COVID-19 vaccination. To make this information useful for clinical practice, patients were grouped into weeks, with for example the first group comprising of patients having had their ^18^F-FDG PET/CT imaging earliest on the same day of vaccination and latest on the sixth day of vaccination group one (days 0–6).

In an immunocompetent patient, inflammatory immunogenic reaction to an external pathogen is expected to be swift [4]. In concordance, our data show that HLA showed the highest incidence in group one (days 0–6) 16/23 (70%). Similarly, the intensity of hypermetabolism measured as rHLA/MBP was highest in this group (rHLA/MBP 2.58 ± 2.1). This is contrary to the study by Cohen et al. [26] which observed a lower incidence during the first 5 days but is in concordance with the study by Kubota et al. in which the highest incidence was observed during the first 5 days [2].

HLA persisted until at least 21 days after vaccination (until group three, (14–20 Days)). The incidence of HLA as well as rHLA/MBP was higher in group three (14–20 days) than in group two (7–13 days): 57% vs. 44%, 5.05 ± 4.33 vs. 3.9 ± 2.81, and 2.32 ± 1.8 vs. 1.83 ± 1.38. This finding may be attributed to various other individual contributing factors such as age, immune status, or immunosuppressive therapy which were not separately assessed in this study [2].

**Figure 5:**
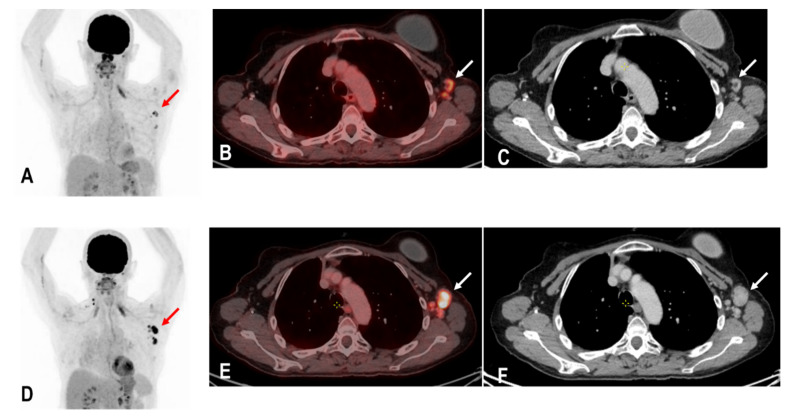
A patient with status post breast carcinoma and hypermetabolic lymph nodes in the ipsilateral lymph nodes of the tumor site (red and white arrows) (**A**–**C**). Unfortunately, the patient also received a COVID-19 vaccination on the same ipsilateral side of tumor involvement, and an ^18^F-FDG PET/CT was performed 25 days following the COVID-19 vaccination. Tumor involvement could not be excluded. A follow-up PET/C 3 months later showed a metabolic and morphological progression of the ipsilateral lymph nodes confirming the diagnosis of tumor involvement rather than HLA (red and white arrows) (**D**–**F**). Due to known tumor involvement, the patient was excluded from the analysis of this study.

All parameters for HLA dropped markedly 21 days after vaccination. There were no significant differences in SUVmax HLA, rHLA/MBP, and rHLA/RL in group four (4,10,22–25 days), group five (29–35 days), and in group six (>35 days). All three assessed parameters for HLA dropped statistically significantly in the subgroup analysis of groups 1–3 (days 0–20) versus groups 4–6 (days 21–80) (Table 4) suggesting that an FDG PET/CT can be performed 21 days post COVID-19 vaccination at the earliest. However, there were a few patients in these groups where HLA persisted, e.g., in group six (35–80 days). Therefore, diagnostic physicians still need to be aware of this pitfall and clinicians are advised not to perform a COVID-19 vaccination on the ipsilateral side of possible tumor involvement (Figure 5), record the date of vaccination carefully, and preferably delay the date of the FDG PET/CT for a minimum of 21 days, and if possible, for about 35 days.

This study has some limitations. First, due to its retrospective nature, we could not assess the impact of the patients’ immune status or the possible influence of ongoing immunosuppressive therapies in view of insufficient clinical data. While some studies have shown that the immune status can affect the characteristics of HLA [4,27], the fact that our results are in line with what was reported in other patient populations [2,26] suggests that the impact on our population may be negligible. Immune response and subsequently HLA is reduced in immunocompromised patients in comparison to immunocompetent patients [4,28,29,30]; therefore, we do not expect that the maximum duration of HLA in our study is underestimated. Second, it was reported that the association between vaccination and HLA is more common in patients younger than 64 years [31]. As our population mostly consists of patients older than 64, the incidence and correlation in younger patients could not be assessed. Finally, the results of the present study only pertain to patients vaccinated with BNT162b2 and mRNA-1273 vaccines. We therefore cannot rule out that the results of the present study may not fit patients injected with other mRNAs- or viral vector-based vaccines.

In conclusion, in the current COVID-19 pandemic and subsequent mass vaccinations, it is crucial for diagnostic physicians to assess the recent history of COVID-19 vaccination prior to a PET/CT scan, including the date and site of vaccination as well as a possible tumor or infectious involvement of the HLA to reduce the risk of false-positive calls. If feasible, an FDG PET should be postponed by approximately 3 weeks after vaccination if an accurate evaluation of axillary status is especially required, for example, in the case of breast cancer or lymphoma.

## Figures and Tables

**Table 1:** Summary of patient characteristics (n = 146).

Demographic Characteristics		
mean age + SD (y)	66.39 ± 12.9	
female	72 (49%)	
male	74 (51%)	
purpose of FDG PET/CT		
staging	91 (62%)	
re-staging	53 (36%)	
non-oncological indication	2 (1%)	
site of vaccination		
left	120 (82%)	
right	26 (18%)	
Clinical diagnosis		
anal carcinoma	4	(2.7%)
appendix carcinoma	1	(0.7%)
breast carcinoma	21	(14.4%)
cholangiocarcinoma	2	(1.4%)
chronic lymphocytic leukemia	1	(0.7%)
colon carcinoma	10	(6.8%)
cancer of unknown primary	5	(3.4%)
esophageal carcinoma	12	(8.2%)
gastric carcinoma	11	(7.5%)
urothelial carcinoma	1	(0.7%)
hepatocellular carcinoma	1	(0.7%)
hypopharyngeal carcinoma	1	(0.7%)
infection	2	(1.4%)
lung carcinoma	30	(20.5%)
lymphoma	25	(17.1%)
melanoma	3	(2.1%)
myeloma	1	(0.7%)
pancreatic carcinoma	6	(4.1%)
rectal carcinoma	6	(4.1%)
pulmonary nodule	1	(0.7%)
thyroid carcinoma	1	(0.7%)
uterine carcinoma	1	(0.7%)

Categorical variables are reported as frequency and percentage. Continuous variables are reported as mean with standard deviation.

**Table 2:** Characteristics of SUVmax HLA, rHLA/MBP, and rHLA/RL according to groups.

Group	Group 1 (0–6 Days)	Group 2 (7–13 Days)	Group 3 (14–20 Days)	Group 4 (21–27 Days)	Group 5 (28–34 Days)	Group 6 (35–80 Days)	Overall (0–80 Days)	*p*-Value
no. of patients	23	32	21	19	15	36	146	
SUVmax HLA	4.97 ± 4.1	3.9 ± 2.81	5.05 ± 4.33	2.25 ± 1.85	1.9 ± 1.17	2.02 ± 1.74	3.35 ± 3.13	<0.001
ratio HLA vs. MBP (rHLA/MBP)	2.58 ± 2.1	1.83 ± 1.38	2.32 ± 1.8	1.07 ± 0.95	0.88 ± 0.56	0.87 ± 0.76	1.59 ± 1.49	<0.001
ratio HLA vs. RL (rHLA/RL)	5.5 ± 4.82	5.41 ± 5.73	6.11 ± 5.99	3.17 ± 3.74	2.25 ± 2.33	2.4 ± 2.02	4.17 ± 4.6	<0.001

Categorical variables are reported as frequency and percentage. Continuous variables are reported as mean with standard deviation. HLA = hypermetabolic lymphadenopathy, MPB = mediastinal blood pool, RL = reference lymph node.

**Table 3:** Characteristics of SUVmax HLA, rHLA/MBP and rHLA/RL groups 1–3 versus groups 4–6.

	Groups 1–3 (0–20 Days)	Groups 4–6 (21–80 Days)	*p*-Value
no. of patients	76	70	
SUVmax HLA	4.54 ± 3.68	2.06 ± 1.65	<0.001
ratio HLA vs. MBP (rHLA/MBP)	2.19 ± 1.74	0.94 ± 0.77	<0.001
ratio HLA vs. RL (rHLA/RL)	5.63 ± 5.48	2.59 ± 2.64	<0.001

**Table 4:** The incidence of HLA defined as the ratio SUVmaxHLA/SUVmaxMBP greater than 1.5 for each group.

	Incidence			
	Present		Absent	
Days after Vaccination (Groups)	N	Row %	N	Row %
group 1 (0–6 days)	16	69.6%	7	30.4%
group 2 (7–13 days)	14	43.8%	18	56.2%
group 3 (14–20 days)	12	57.1%	9	42.9%
group 4 (21–27 days)	4	21.1%	15	78.9%
group 5 (28–34 days)	4	26.7%	11	73.3%
group 6 (35–80 days)	4	11.1%	32	88.9%
overall (0–80 days)	54	37%	92	63%

**Table 5:** Characteristics of HLA and sequence of vaccination.

Group 1 (0–6 Days) (n = 23)	First Shot	Second Shot	Third Shot	*p*-Value
incidence	3/6 (50%)	4/5 (80%)	9/12 (75%)	
SUVmax HLA	5.53 ± 2.47	6.75 ± 4.81	3.95 ± 2.75	0.42
rHLA/MBP	2.65 ± 2.47	3.29 ± 2.71	2.24 ± 1.67	0.65
rHLA/RL	4.82 ± 4.92	8.37 ± 6.90	4.64 ± 3.62	0.33
Group 2 (7–13 days) (n = 32)	First shot	Second shot	Third shot	
incidence	3/4 (75%)	10/19 (53%)	1/9 (11%)	
SUVmax HLA	4.83 ± 3.74	4.46 ± 2.90	2.29 ± 1.57	0.13
rHLA/MBP	2.26 ± 1.65	2.12 ± 1.46	1.03 ± 0.77	0.13
rHLA/RL	7.70 ± 5.45	6.29 ± 6.65	2.54 ± 1.71	0.19
Group 3 (14–20 days) (n = 21)	First shot	Second shot	Third shot	
incidence	9/14 (64%)	3/5 (60%)	0/2 (0%)	
SUVmax HLA	5.50 ± 4.89	4.31 ± 3.67	3.7 ± 1.69	0.8
rHLA/MBP	2.56 ± 2.02	1.79 ± 1.45	1.95 ± 0.77	0.7
rHLA/RL	6.67 ± 7.12	4.81 ± 3.21	5.44 ± 2.34	0.84
Group 4 (21–27 days) (n = 19)	First shot	Second shot	Third shot	
incidence	1/8 (13%)	3/8 (38%)	0/3 (0%)	
SUVmax HLA	2.23 ± 2.08	2.72 ± 1.86	1.04 ± 0.58	0.43
rHLA/MBP	0.95 ± 0.79	1.46 ± 1.13	0.38 ± 0.23	0.23
rHLA/RL	2.77 ± 2.84	4.17 ± 5.04	1.60 ± 0.53	0.58
Group 5 (28–34 days) (n = 15)	First shot	Second shot	Third shot	
incidence	1/4 (25%)	3/8 (38%)	0/3 (0%)	
SUVmax HLA	1.45 ± 1.21	2.49 ± 1.08	1.1 ± 0.78	0.13
rHLA/MBP	0.62 ± 0.55	1.15 ± 0.55	0.52 ± 0.34	0.14
rHLA/RL	2.66 ± 1.03	2.77 ± 3.02	0.81 ± 0.21	0.47
Group 6 (35–80 days) (n = 36)	First shot	Second shot	Third shot	
incidence	0/1 (0%)	4/24 (14%)	0/11 (0%)	
SUVmax HLA	N/A	2.17 ± 1.92	1.31 ± 0.66	0.48
rHLA/MBP	N/A	0.96 ± 0.83	0.61 ± 0.32	0.56
rHLA/RL	N/A	2.58 ± 2.22	1.59 ± 0.83	0.52
Overall (0–80 days) (n = 146)	First shot	Second shot	Third shot	
incidence	17/37 (46%)	27/73 (37%)	10/36 (27%)	
SUVmax HLA	4.21 ± 4.25	3.32 ± 2.79	2.53 ± 2.14	0.07
rHLA/MBP	1.94 ± 1.84	1.55 ± 1.39	1.30 ± 1.27	0.18
rHLA/RL	5.08 ± 5.44	4.29 ± 4.80	2.99 ± 2.71	0.14

Categorical variables are reported as frequency and percentage. Continuous variables are reported as mean with standard deviation. MPB = mediastinal blood pool, and RL = reference lymph node. *p*-values < 0.05 were considered significantly different.

## Data Availability

All reviewed imaging modalities were performed during clinical routine. Patient data are stored in the local archiving system at the University Hospital Mainz, Germany, and St. Claraspital, Switzerland.
